# Uptake, willingness, and practices toward seasonal influenza vaccination among healthcare workers in China: a cross-sectional study

**DOI:** 10.3389/fpubh.2026.1682897

**Published:** 2026-02-12

**Authors:** Pei Zhang, Shanhong Fan, Ling Lv, Xinxin Huo, Jie Zhao

**Affiliations:** Department of Disease Control and Prevention, Tangdu Hospital, Fourth Military Medical University, Xi’an, Shaanxi, China

**Keywords:** China, healthcare workers, influenza vaccine, practice, public health interventions, uptake, willingness

## Abstract

**Background:**

Seasonal influenza remains a major public health burden globally, with healthcare workers (HCWs) at heightened risk due to occupational exposure and potential for nosocomial transmission. Despite this, influenza vaccination rates among HCWs in China remain suboptimal.

**Objectives:**

To assess the uptake, willingness, and influencing factors related to seasonal influenza vaccination among HCWs in a major tertiary hospital in Xi’an, China, during the post-COVID-19 era.

**Methods:**

A cross-sectional, single-center survey was conducted in December 2023 among 1,196 HCWs at Tangdu Hospital (Xi’an, Shaanxi Province). Data were collected via a validated, anonymous online questionnaire covering demographics, vaccination history, attitudes, and barriers. Multivariate logistic regression was used to identify factors associated with vaccination uptake and willingness.

**Results:**

Only 19.65% of HCWs reported receiving at least one influenza vaccination in the past 2 years. Vaccination rates declined significantly from 2022 to 2023. In multivariable models, influenza vaccine uptake was positively associated with household vaccination rates (adjusted OR = 7.89, 95% CI: 5.61–11.09, *p* < 0.001), willingness to be vaccinated (adjusted OR = 1.51, 95% CI: 1.11–2.07, *p* = 0.009). Willingness to be vaccinated was higher among HCWs with recent flu-like symptoms (adjusted OR = 1.52, 95% CI: 1.16–1.99, *p* = 0.002) and lower among female HCWs (adjusted OR = 0.58, 95% CI: 0.41–0.82, *p* = 0.002).

**Conclusion:**

Despite positive attitudes, influenza vaccine uptake among Chinese HCWs remains low, with signs of post-pandemic decline. Addressing safety concerns, improving access, and targeted education may improve coverage and strengthen healthcare system resilience.

## 1 Introduction

The influenza A and B viruses are the primary causes of seasonal influenza, a type of acute viral respiratory infection. Even in the post-COVID-19 era, it remains a significant global health burden. The World Health Organization (WHO) estimates that influenza causes between 3 and 5 million cases of severe illness and between 290,000 and 650,000 respiratory deaths globally each year (1, 2). In healthcare settings, where patients and healthcare workers (HCWs) are at higher risk, the burden is especially noticeable. In addition to being vulnerable to influenza infection from their work, HCWs are also at risk of nosocomial transmission, especially to high-risk patient groups like the older adults, children, and people with compromised immune systems (3).

Influenza vaccination has long been recognized as the most effective strategy for preventing influenza infection and reducing its complications (4). Annual influenza vaccination for HCWs lowers absenteeism due to illness, increases productivity, and above all, protects vulnerable patients from preventable harm (5). As a result, many countries such as the United States, Canada, and several European Union nations have implemented strict guidelines or even laws requiring annual influenza vaccinations for HCWs (6). However, there are still disparities in influenza vaccination coverage across the globe, particularly in low- and middle-income countries (LMICs), where there are still financial, logistical, and perceptional obstacles (7).

The National Immunization Program (NIP) in China does not include influenza vaccination, and the resulting out-of-pocket expenses and decentralized policy have resulted in less than ideal uptake. Adult influenza vaccination coverage nationwide was only 0.62% during the 2018–2019 season (2), but it rose marginally in the years following the COVID-19 pandemic to roughly 3% in the following years (8, 9). Vaccination rates among HCWs in particular are still surprisingly low; estimates vary by region and institutional policy, ranging from 5 to 18% (10–13). This is in stark contrast to HCWs in high-income countries, where coverage usually exceeds 75% (14).

Early in 2020, the COVID-19 pandemic broke out, drastically changing public perceptions of immunization and infectious disease prevention. According to a number of studies, attitudes toward other vaccines, including influenza vaccines, may have improved as a result of the pandemic’s increased awareness of respiratory infections and the development of mass immunization infrastructure (15). On the other hand, some subpopulations may have been hesitant due to vaccine fatigue, false information, and logistical difficulties during the pandemic (16). HCWs, who serve as both recipients of immunization programs and essential disseminators of vaccine-related information to the general public, should pay special attention to understanding these changes.

Vaccine hesitancy, which the WHO defines as the delay in accepting or refusing vaccines despite their availability, is one of the main obstacles to increasing influenza vaccination coverage (17). Complacency, convenience, and confidence, the so-called “3Cs” model, are the three main factors that contribute to vaccine hesitancy (18). HCWs’ hesitancy is especially concerning in healthcare settings. In addition to providing direct patient care, healthcare workers also act as role models for vaccine compliance. Patients’ trust and vaccination decisions are directly impacted by HCWs’ attitudes and practices (19, 20). Studies have shown that when HCWs express doubts about vaccine safety or necessity, it can hinder broader public health initiatives (21).

From a behavioral science perspective, influenza vaccination among HCWs can be understood using established frameworks that explain why individuals do or do not adopt preventive health behaviors. The WHO ‘3Cs’ model (confidence, complacency, convenience) highlights trust in vaccines and systems, perceived need for vaccination, and practical access constraints as core drivers of vaccine hesitancy. Complementary health behavior theories, such as the Health Belief Model (HBM) and the Theory of Planned Behavior (TPB) (22), further clarify how risk perceptions (e.g., perceived susceptibility and severity), perceived benefits and barriers, cues to action, and social norms shape vaccination intention and, ultimately, uptake. These frameworks provide a theoretical rationale for jointly assessing both vaccination behavior (uptake) and intention (willingness), as well as specific barriers and facilitators that can be targeted through policy, organizational, and communication interventions.

The body of research examining HCWs’ knowledge, attitudes, and practices (KAP) regarding viral infection vaccinations, including COVID-19 (23, 24) and influenza has expanded in recent years (25, 26). Nonetheless, the majority of this research has been carried out in high-income settings. High-quality data analyzing the sociodemographic and occupational factors influencing vaccination uptake and hesitancy among health care HCWs is still lacking in China, particularly in a tertiary hospital setting. The post-COVID-19 context offers a unique opportunity to explore how these dynamics have shifted and what barriers remain to achieving optimal coverage.

To address this gap, we carried out a cross-sectional survey in December 2023 among HCWs at a major medical institution in the Tangdu Hospital (Xi’an, Shaanxi Province). Guided by behavioral frameworks of vaccine decision-making (e.g., 3Cs model: confidence–complacency–convenience and health behavior theories emphasizing risk perception, social norms, and access constraints), the main objectives were to quantify HCWs’ influenza vaccine uptake during and after the COVID-19 pandemic, assess willingness (intention) to vaccinate, and identify key facilitators and barriers that may be targeted to improve coverage. The questionnaire used in this study was adjusted for the local context based on previously validated studies (27–30). This study would add to the small body of research in LMICs by examining the complex factors that influence HCWs’ influenza vaccination behavior in a Chinese tertiary hospital setting. It also provides evidence-based recommendations for developing focused educational, institutional, and policy-level interventions. In the post-COVID era, the results can also guide more comprehensive plans to raise adult vaccination rates and prepare for pandemics.

## 2 Methods

### 2.1 Study design and population

This cross-sectional, single-center survey was conducted among healthcare workers (HCWs) at Tangdu Hospital, located in Xi’an, Shaanxi Province. The total number of healthcare workers at this institute was estimated to exceed 3,000. For the purpose of this study, *healthcare workers (HCWs)* were defined as all staff working at Tangdu Hospital whose routine duties involve direct patient care, diagnostic/therapeutic support services, infection prevention/disease-control functions, or administrative/operational support within clinical services. Accordingly, eligible participants included nurses, medical professionals (physicians), medical technicians (including laboratory and radiology staff), pharmacists, healthcare recruiters, managers/administrative staff, researchers, project-based staff, and other allied personnel. In this tertiary-hospital setting, HCWs may also differ by employment arrangement (e.g., formally employed vs. contract/project-based); while we captured occupational role and professional title, employment contract type was not collected as a standalone variable and is therefore discussed as a potential source of heterogeneity. The data collection period spanned from December 3, 2023, to December 31, 2023 in which 1,196 completed the survey questionnaires.

### 2.2 Data collection and study measures

Data were collected using an anonymous self-administered, online questionnaire, completed voluntarily by participants. This pre-designed questionnaire was extracted and adapted from previous similar studies (27–30) and further developed specifically for the purpose of this study. Since all questions were mandatory on the online platform, the recorded data was complete with no missing values. Duplicate entries were excluded from the final analysis.

The instrument was adapted from prior influenza vaccination surveys among HCWs and related KAP research (27–30) to ensure coverage of determinants that have been repeatedly linked to influenza vaccine uptake and intention. In addition, item selection and grouping were aligned with behavioral frameworks of vaccine decision-making, particularly the WHO ‘3Cs’ (confidence, complacency, convenience) and health behavior theory constructs (e.g., risk perception, cues to action, and social norms). Accordingly, items assessing concerns about safety/side effects and perceived effectiveness capture *confidence*; items assessing perceived necessity and perceived mildness of influenza capture *complacency*; and items assessing access/availability and process-related barriers capture *convenience*. Household vaccination and peer encouragement items reflect normative/social influence, and recent flu-like symptoms were included as a potential cue-to-action and proxy for perceived susceptibility.

The questionnaire was composed of four sections (Part I–IV). The four-part structure was designed to capture (I) background characteristics, (II) clinical/household context, (III) recent influenza-like illness and work practices relevant to occupational exposure and presenteeism, and (IV) vaccination behavior (uptake), intention (willingness), and specific facilitators/barriers and improvement suggestions.

Part I showed the socio-demographic characteristics including age (years), gender (female or male), highest educational level (junior high school or below, senior high school/technical secondary school, junior college, bachelor’s degree, master’s degree, doctorate or above), job (nurse, medical professional, medical technician, healthcare recruiter, manager, pharmacist, researcher, project-based staff, or other), professional title (primary, middle, senior or others), and department/division (neurosurgery, thoracic surgery, pediatrics, functional department, orthopedics, cardiovascular medicine, hospital affairs office, gastroenterology, diagnostic radiology, others). Because employment contract status (e.g., formally employed vs. contract-based) was not measured separately, we report the distribution of HCWs by job category and professional title and discuss how differing employment arrangements may influence access and vaccination behavior.

Part II showed information about the clinical characteristics including individuals’ medical history [yes (including hypertension, hyperlipidemia, chronic bronchitis, diabetes, malignant tumors, hypothyroidism and others) or no], history of immunosuppressive therapy (during the past 6 months), number of people living with them, and those who have received the flu vaccine in the last two influenza seasons.

Furthermore, in part III, flu-like symptoms [yes (including fever, cough, sore throat) or no- during last 3 months] were recorded. In case of any positive response regarding the flu-like symptoms, the highest body temperature, the final diagnosis (common cold, influenza, mycoplasma pneumonia, COVID-19, or unknown), method of diagnosis (clinical diagnosis, nucleic acid test, serum specific antibody test, or undiagnosed), and going on a work leave [yes (duration) or no] were recoded. In case of not going on a work leave, information regarding the types of masks when having flu-like symptoms at work (disposable masks, disposable surgical masks, N95 masks, not wearing a mask or others) and the reasons for not leaving work, such as (1) concerns about the increased workload of colleagues, (2) concerns about salary or performance deduction, (3) concerns about the continuity of treatment or care, (4) the condition was mild and did not affect work, (5) concerns about their boss’s opinion, (6) concerns about being rejected by colleagues, (7) concerns about their patient’s condition and other, were collected.

In part IV, we obtained the HCWs’ vaccination status of participants over the last 2 years (2022, 2023) and the reasons for getting/not getting the flu vaccine, as well as their willingness for vaccination in the next flu season. The questionnaire ended with an open-ended question on the measures proposed by HCWs on how to improve influenza vaccination coverage in their work environment.

### 2.3 Ethical considerations

The study protocol were approved by the Research Ethics Committee of the Fourth Military Medical University. The participants were reassured of the confidentiality of the collected information and signed an informed consent form.

### 2.4 Statistical analysis

All data were analyzed using SPSS version 26.0 (IBM Corporation, Armonk, NY, United States). Descriptive statistics were used to summarize participants’ demographic and clinical characteristics, as well as vaccination status and behavior.

Categorical variables were compared using the chi-squared test, and continuous variables were assessed using the independent sample t-test. Multivariable logistic regression analysis was conducted to identify factors associated with vaccination uptake and willingness to be vaccinated. To reduce sparse cells and improve the stability of categorical comparisons, we collapsed low-frequency categories prior to analysis. For multivariable logistic regression, educational level was analyzed as ‘university and above’ (bachelor’s/master’s/doctorate) versus ‘below’, and job was analyzed as ‘direct contact’ versus ‘indirect contact’ with patients. Odds ratios (ORs) and 95% confidence intervals (CIs) were reported. All statistical tests were two-sided, and *p*-values < 0.05 were considered statistically significant.

## 3 Results

### 3.1 Demographic and occupational characteristics

A total of 1,196 HCWs were included in this study, of whom 235 (19.65%) reported receiving at least one influenza vaccination in the past 2 years, and 961 (80.35%) were unvaccinated.

The mean age of participants was 37.21(8.21) years. Vaccinated HCWs were slightly older than unvaccinated HCWs (38.29 (9.29) vs. 36.94 (7.9) years, *p* = 0.042). In the total population, the majority were female (*n* = 991, 82.86%) and held a bachelor’s degree (*n* = 848, 70.9%), with no significant difference in gender or education between groups (*p* = 0.66, *p* = 0.165, respectively). The sample included both frontline clinical and support roles. The majority of participants were nurses (*n* = 672, 56.19%) and medical professionals (*n* = 196, 16.39%), followed by medical technicians (9.87%) and pharmacists (2.34%). Non-clinical/support roles (healthcare recruiters, managers, researchers, project-based staff, and ‘other’) comprised 15.22% of respondents, with no statistically significant variation across job categories (*p* = 0.423). However, a statistically significant difference in vaccination status was observed based on professional title (*p* = 0.014), with a higher proportion of vaccinated participants holding senior titles (*n* = 24, 10.21%) compared to unvaccinated (*n* = 52, 5.41%). Participants represented a wide range of hospital departments, including neurosurgery (*n* = 63, 5.27%), thoracic surgery (*n* = 60, 5.02%), pediatrics (*n* = 49, 4.1%), and others, with no significant difference in vaccination status across departments (*p* = 0.487) (Table 1).

**Table 1:** Demographic and occupational characteristics of participants.

Variables	No vaccination (*n* = 961)	At least one vaccination (*n* = 235)	Total (*n* = 1,196)	*p*-value^c^
Age (years)	36.94 (7.9)^a^	38.29 (9.29)	37.21 (8.21)	**0.042**
Gender				
Female	794 (82.62%)^b^	197 (83.83%)	991 (82.86%)	0.66
Male	167 (17.38%)	38 (16.17%)	205 (17.14%)	
Highest educational level				
Junior high school or below	44 (4.58%)	4 (1.7%)	48 (4.01%)	0.154
Senior high school / technical secondary school or Junior college	65 (6.77%)	15 (6.38%)	80 (6.69%)	
Bachelor’s degree	671 (69.82%)	177 (75.32%)	848 (70.9%)	
Master’s degree or above	181 (18.83%)	39 (16.59%)	220 (18.4%)	
Job				
Nurse	537 (55.88%)	135 (57.45%)	672 (56.19%)	0.360
Medical professional	161 (16.75%)	35 (14.89%)	196 (16.39%)	
Medical technician	88 (9.16%)	30 (12.77%)	118 (9.87%)	
Healthcare Recruiter	52 (5.41%)	9 (3.83%)	61 (5.1%)	
Other	123 (12.8%)	26 (11.07%)	149 (12.46%)	
Professional title^c^				
Primary	404 (42.04%)	91 (38.72%)	495 (41.39%)	**0.014**
Middle	387 (40.27%)	101 (42.98%)	488 (40.8%)	
Senior	52 (5.41%)	24 (10.21%)	76 (6.35%)	
Others	118 (12.28%)	19 (8.09%)	137 (11.45%)	
Department/division				
Neurosurgery	52 (5.41%)	11 (4.68%)	63 (5.27%)	0.487
Thoracic Surgery	48 (4.99%)	12 (5.11%)	60 (5.02%)	
Pediatrics	45 (4.68%)	4 (1.7%)	49 (4.1%)	
Administrative department	35 (3.64%)	13 (5.53%)	48 (4.01%)	
Orthopedics	36 (3.75%)	11 (4.68%)	47 (3.93%)	
Cardiovascular medicine	35 (3.64%)	7 (2.98%)	42 (3.51%)	
Hospital affairs office	34 (3.54%)	7 (2.98%)	41 (3.43%)	
Gastroenterology	32 (3.33%)	9 (3.83%)	41 (3.43%)	
Diagnostic radiology	31 (3.23%)	9 (3.83%)	40 (3.34%)	
Others	613 (63.79%)	152 (64.68%)	765 (63.69%)	

^a^Shown in mean (SD). ^b^Shown in count (percentage). ^c^*p*-values were calculated using the t-test and chi-square test. ^d^Primary: equals a resident physician; middle: equals chief physician; senior: equals professor. Bold values show the statistically significant results.

### 3.2 Clinical characteristics and vaccination context

As shown in Table 2, the prevalence of comorbidities was generally low across the cohort, and no significant differences were observed in medical history or immunosuppressive therapy between the groups. The number of vaccinated cohabitants differed significantly between groups; vaccinated participants reported a mean of 1.79 vaccinated cohabitants, while unvaccinated participants reported 0.59 (*p* < 0.001). Similarly, the perceived community vaccination rate was significantly higher among vaccinated HCWs (55.61% vs. 17.96%, *p* < 0.001). Participants’ willingness to be vaccinated showed significant variation by vaccine status (*p* = 0.004). Among vaccinated HCWs, 44.26% expressed willingness to continue vaccination, compared to 32.78% in the unvaccinated group.

**Table 2:** Clinical characteristics of participants and vaccination context.

Variables	No vaccination (*n* = 961)	At least one vaccination (*n* = 235)	Total (*n* = 1,196)	*p*-value^a^
Medical history				
No	860 (89.49%)	203 (86.38%)	1,063 (88.87%)	0.073
Hypertension	40 (4.16%)	10 (4.25%)	50 (4.18%)	
Hyperlipidemia	24 (2.49%)	4 (1.7%)	28 (2.34%)	
Chronic bronchitis	12 (1.24%)	5 (2.12%)	17 (1.42%)	
Diabetes	12 (1.24%)	2 (0.85%)	14 (1.17%)	
Malignant tumors	4 (0.41%)	1 (0.42%)	5 (0.41%)	
Hypothyroidism	6 (0.62%)	1 (0.42%)	7 (0.58%)	
Others	20 (2.08%)	12 (5.1%)	32 (2.67%)	
History of immunosuppressive therapy (last 6 months)				
Yes	8 (0.83%)	4 (1.7%)	12 (1%)	0.267
No	953 (99.16%)	231 (98.3%)	1,184 (99%)	
Number of people living with	3.25 (1.25)	3.21 (1.39)	3.24 (1.48)	0.742
Number of people living with those who have received the flu vaccine	0.59 (1.03)	1.79 (1.5)	0.82 (1.23)	**<0.001**
The percentage of vaccination among people around	17.96 (30.52)	55.61 (40.59)	25.31 (35.69)	**<0.001**
Flu-like symptoms (last 3 months)				
Yes	569 (59.21%)	135 (57.45%)	704 (58.86%)	0.623
No	392 (40.79%)	100 (42.55%)	492 (41.14%)	
Number of flu vaccinations during (last 2 years)				
0	961 (100%)	0	961 (80.35%)	**<0.001**
1	0	167 (71.06%)	167 (13.96%)	
2	0	68 (28.94%)	68 (5.69%)	
Willingness to be vaccinated				
Yes	315 (32.78%)	104 (44.26%)	419 (35.03%)	**0.004**
Unsure	308 (32.05%)	61 (25.96%)	369 (30.85%)	
No	338 (35.17%)	70 (29.79%)	408 (34.11%)	

^a^*p*-values were calculated using the t-test and chi-square test. Bold values show the statistically significant results.

### 3.3 Influenza-like symptoms and work practices

Among participants who reported flu-like symptoms in the past 3 months (n = 704), no statistically significant differences were found in symptoms (e.g., fever, cough, sore throat) or diagnosis methods between groups. However, the duration of work leave differed significantly: vaccinated participants took fewer days off compared to unvaccinated participants [1.64 (1.21) vs. 3.4 (4.74) days, respectively, *p* = 0.024]. Despite symptoms, 91.34% of HCWs did not take work leave, with “concerns about increased workload for colleagues” more frequently cited by vaccinated HCWs (27.41% vs. 8.66%, *p* = 0.036). Most HCWs reported using disposable surgical masks (68.04%) while symptomatic at work, with no differences between groups (Table 3).

**Table 3:** History of getting flu-like symptoms and related information.

Variables	No vaccination (*n* = 569)	At least one vaccination (*n* = 135)	Total (*n* = 704)	*p*-value^a^
Flu-like symptoms (last 3 months)				
Fever	322 (56.59%)	75 (55.55%)	397 (%)	0.926
Cough	445 (78.2%)	110 (81.48%)	555 (%)	
Sore throat	428 (75.21%)	101 (74.81%)	529 (%)	
Highest body temperature	38.49 (0.88)	38.31 (2.5)	38.45 (1.36)	0.320
The final diagnosis				
Common cold	188 (33.04%)	40 (29.62%)	228 (%)	0.393
Influenza	107 (18.8%)	31 (22.96%)	138 (%)	
Mycoplasma pneumonia	41 (7.2%)	7 (5.18%)	48 (%)	
COVID-19	26 (4.56%)	10 (7.4%)	36 (%)	
Unknown	236 (41.47%)	51 (37.77%)	287 (%)	
Method of diagnosis				
Clinical diagnosis	131 (23.02%)	31 (22.96%)	144 (%)	**<0.001**
Nucleic acid test	45 (7.9%)	16 (11.85%)	61 (%)	
Serum-specific antibody test	36 (6.32%)	3 (2.22%)	39 (%)	
Undiagnosed	385 (67.66%)	90 (66.66%)	475 (%)	
Go on a work leave				
Yes	50 (8.79%)	11 (8.15%)	61 (8.66%)	0.812
No	519 (91.21%)	124 (91.85%)	643 (91.34%)	
Work leave duration (days)	3.4 (4.74)	1.64 (1.21)	3.08 (4.36)	**0.024**
Types of masks when having flu symptoms at work				
Disposable masks	29 (5.1%)	7 (5.19%)	36 (5.11%)	0.994
Disposable surgical masks	388 (68.19%)	91 (67.41%)	479 (68.04%)	
N95 masks	94 (16.52%)	24 (17.78%)	118 (16.76%)	
Not wearing a mask	6 (1.05%)	1 (0.74%)	7 (0.99%)	
Other	52 (9.14%)	12 (8.89%)	64 (9.09%)	
Reasons for not leaving work (among those who did not go on leave)				
Concerns about the increased workload of colleagues	45 (8.66%)	34 (27.41%)	79 (12.27%)	**0.036**
Concerns about salary or performance deduction	35 (6.74%)	15 (12.09%)	50 (7.77%)	
Concerns about the continuity of treatment or care	22 (4.23%)	6 (4.83%)	28 (4.35%)	
The condition was mild and did not affect work	20 (3.85%)	5 (4.03%)	25 (3.88%)	
Concerns about their boss’s opinion	17 (3.27%)	4 (3.22%)	21 (3.26%)	
Concerns about being rejected by colleagues	9 (1.73%)	5 (4.03%)	14 (2.17%)	
Concerns about their patient’s condition	6 (1.15%)	4 (3.22%)	10 (1.55%)	
Other	9 (1.73%)	1 (0.8%)	10 (1.55%)	

^a^p-values were calculated using the t-test and chi-square test. Bold values show the statistically significant results.

### 3.4 Vaccination status and patterns

**Figure 1:**
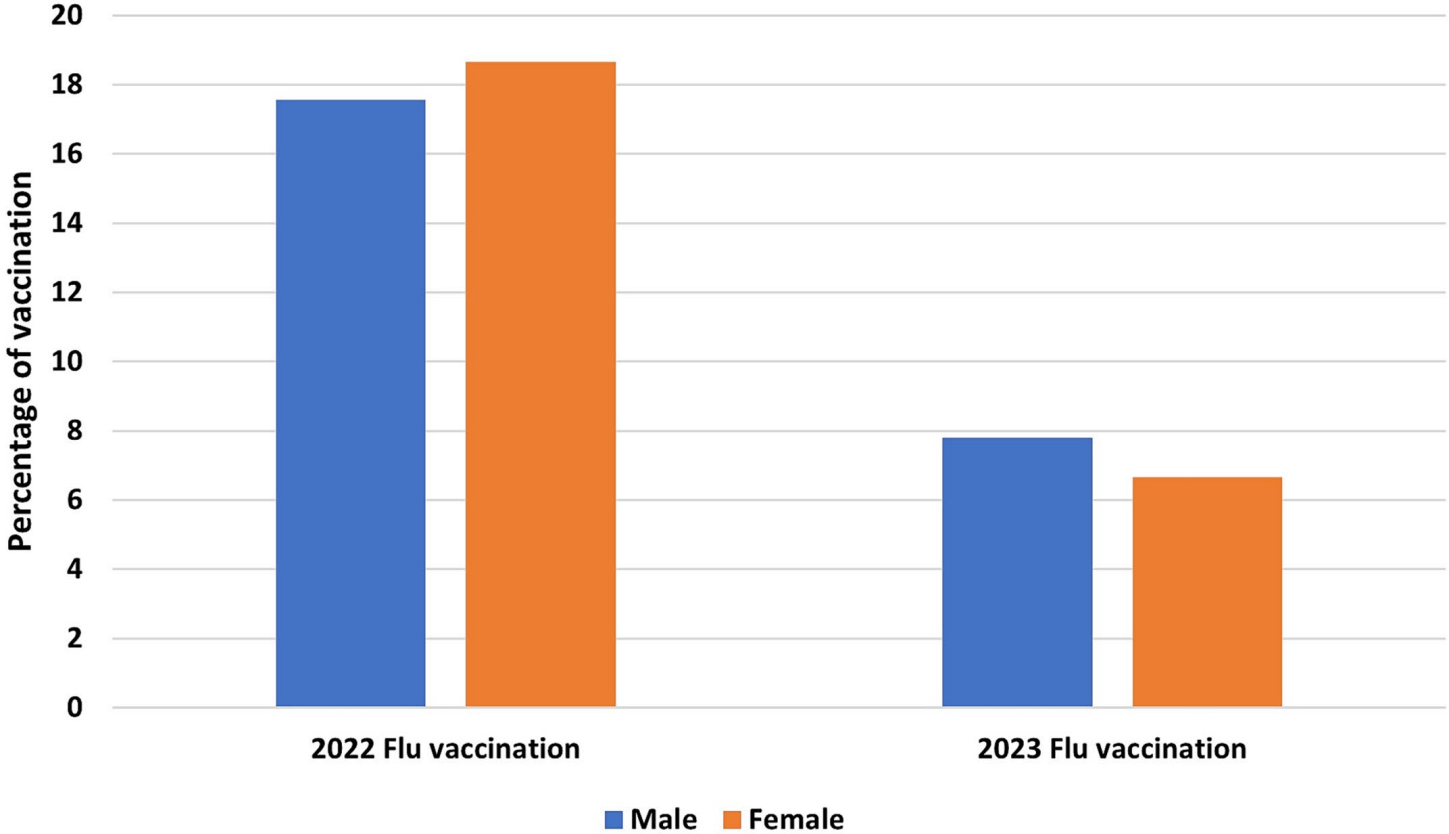
Health care workers’ influenza vaccination status.

As shown in Figure 1, influenza vaccination coverage among HCWs was 17.56, and 18.67% in males and females, respectively, in the 2022 season and decreased to 7.8 and 6.66% in males and females, respectively, in 2023, indicating a significant decrease following the termination of the COVID-19 pandemic (*p* < 0.001).

### 3.5 Motivations and barriers

**Figure 2:**
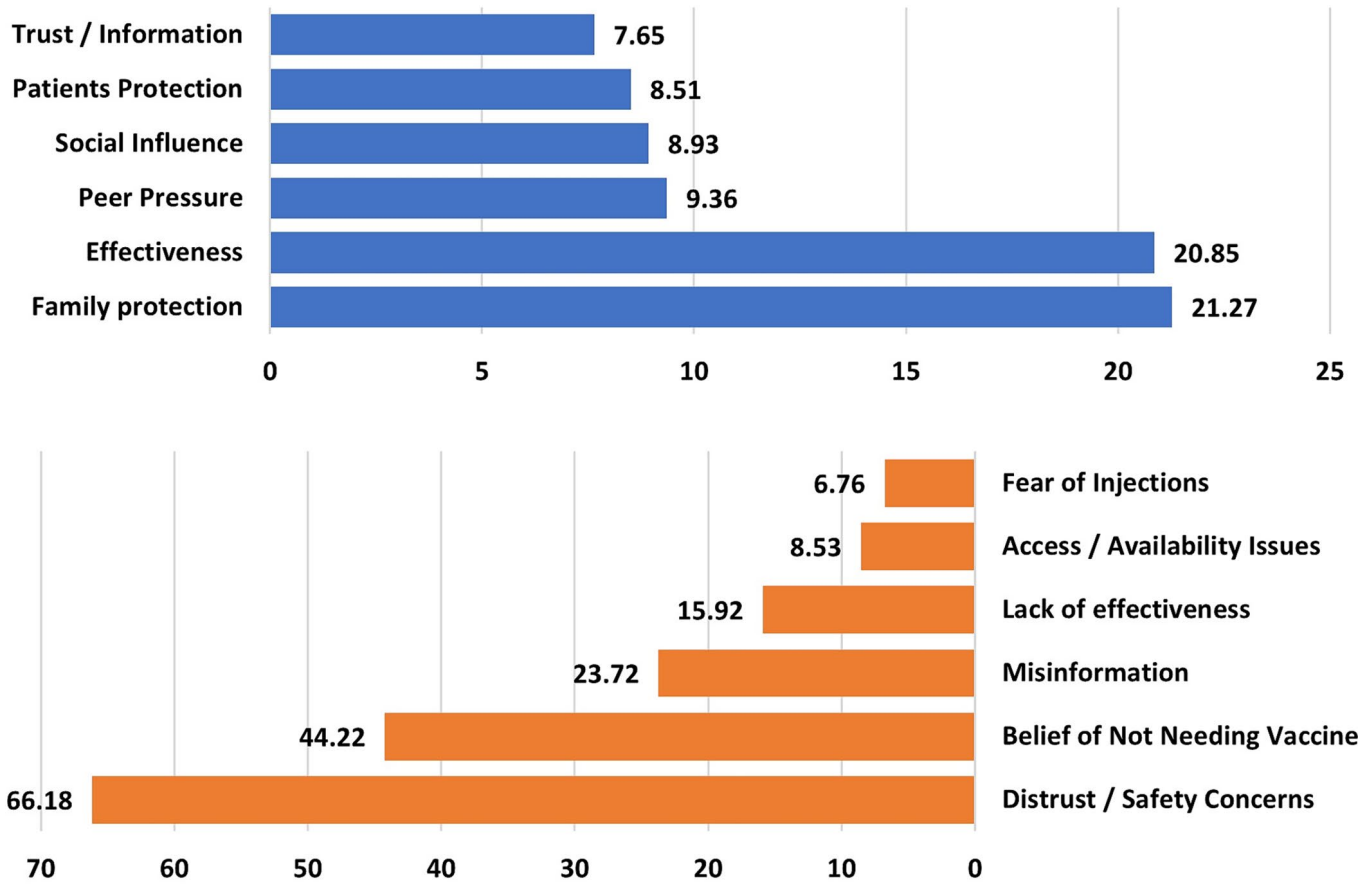
Reasons for taking (top) vs. not taking the flu vaccine (bottom).

As illustrated in Figure 2 and detailed in Table 4, the primary motivations for vaccination included family protection (21.27%), belief in vaccine effectiveness (20.85%), and peer encouragement (9.36%). In contrast, the main reasons for vaccine hesitancy included distrust/safety concerns (66.18%), belief that they do not need the vaccine (44.22%), and perceiving influenza as a minor illness (23.72%). Other cited concerns included the belief in a lack of effectiveness (15.92%), access issues (8.53%), and fear of injections (6.76%).

**Table 4:** Reasons for getting and not getting the vaccine.

Statements	Overarching title	Count (percentage within group)
Reasons for getting the vaccine (Vaccinated respondents, *n* = 235)		
To protect my family members	Family protection	50 (21.27%)
The Flu vaccine is effective	Effectiveness	49 (20.85%)
Someone around me was vaccinated and encouraged me to do so	Peer Pressure	22 (9.36%)
The flu vaccine was promoted through the internet and the media	Social Influence	21 (8.93%)
To protect the patients	Patients Protection	20 (8.51%)
Get vaccinated after consulting the medical staff	Trust/information	18 (7.65%)
Reasons for not getting the vaccine (Unvaccinated respondents, n = 961)		
Afraid of trouble, worried about adverse reactions, not confident about the vaccine, not trusting the vaccine, hesitant about whether to get it, pregnancy/breastfeeding	Distrust/safety concerns	636 (66.18%)
I am strong and do not need a vaccination. I can handle it myself	Belief in not needing a vaccine	425 (44.22%)
Do not be afraid if I get it, since the flu is a minor illness	Misinformation	228 (23.72%)
Vaccines are useless and have no effect	Lack of effectiveness	153 (15.92%)
Cost is a bit expensive, vaccines are not available/no appointment, no time to vaccinate	Access/availability issues	82 (8.53%)
Afraid of injections	Fear of injections	65 (6.76%)

### 3.6 Suggestions to improve influenza vaccination uptake among HCWs

To better understand how influenza vaccination coverage could be improved, participants were asked to provide open-ended suggestions (Table 5). Then all suggestions were categorized under the specified categories. The most frequently cited category was the need to improve access and logistics, reported by 25.94% of respondents. Specific suggestions included optimizing appointment systems, adding vaccination windows, reducing wait times, and enabling more flexible schedules. Some (15.67%) recommended streamlining the vaccination process, such as offering department-based delivery, batch vaccinations, or mobile units to increase convenience. Communication and publicity were also highlighted. 14.59% of HCWs suggested enhancing awareness through early notice, stronger publicity campaigns, and more effective messaging. Interestingly, 14.05% of participants simply expressed general support or encouragement for vaccination programs, while 11.89% requested free or subsidized vaccines for staff or family members. Other suggestions included enhancing transparency about vaccine safety and side effects (9.72%) and expanding vaccination eligibility to family members, including spouses and children (8.1%). These findings emphasize that both system-level improvements (e.g., logistics, delivery models) and behavioral interventions (e.g., targeted communication, education) are likely to be necessary to improve uptake (Table 5).

**Table 5:** Ways to promote influenza vaccination among healthcare workers.

Category	Description	Count	Percent
Improve access and logistics	Suggestions to optimize appointment systems, reduce waiting times, add vaccination staff/windows, allow more flexible schedules.	240	25.94%
Vaccination process improvements	Calls for streamlined or simplified procedures, department-based delivery, mobile services, or batch organization.	145	15.67%
Communication and publicity	Suggestions to enhance awareness via early notices, campaigns, and clearer communication.	135	14.59%
General support/encouragement	General expressions of approval or encouragement	130	14.05%
Free or subsidized vaccination	Requests for free vaccines for staff/families or financial support like discounts.	110	11.89%
Knowledge, safety, and transparency	Requests for info on vaccine safety, side effects, benefits, and monitoring adverse reactions.	90	9.72%
Family inclusion	Calls to include spouses, children, or extended family in vaccination programs.	75	8.1%

### 3.7 Association between socio-demographic and clinical factors and influenza vaccine uptake and willingness

Table 6 presents the unadjusted and adjusted odds ratios (ORs) for the association between socio-demographic and clinical characteristics and both influenza vaccine uptake and willingness to be vaccinated. After adjusting for age, gender, educational level, and professional title, several key associations emerged. The vaccination rate among people living with the respondent was a strong predictor of vaccine uptake. HCWs who reported that ≥50% of their cohabitants were vaccinated had significantly higher odds of receiving the influenza vaccine themselves (adjusted OR: 7.89, 95% CI: 5.61–11.09, *p* < 0.001), indicating strong peer influence within the household. Additionally, participants who reported a willingness to be vaccinated were significantly more likely to have already received the vaccine (adjusted OR: 1.51, 95% CI: 1.11–2.07, *p* = 0.009). Moreover, HCWs with senior professional titles were more likely to have been vaccinated (adjusted OR: 1.99, 95% CI: 1.12–3.52, *p* = 0.018).

**Table 6:** The association of socio-demographic and clinical factors with influenza vaccine uptake and willingness to be vaccinated.

Characteristics	Influenza vaccine uptake				Willingness to be vaccinated			
	Unadjusted OR	*p*-value	Adjusted OR^a^	*p*-value	Unadjusted OR	*p*-value	Adjusted OR^a^	*p*-value
Age (≥30 years to <30 years *)	1.040 (0.698–1.551)	0.847	1.037 (0.679–1.582)	0.867	0.761 (0.551–1.050)	0.097	0.719 (0.510–1.013)	0.060
Gender (Female to male*)	1.090 (0.742–1.603)	0.660	1.531 (0.967–2.424)	0.069	**0.487 (0.359–0.660)**	**<0.001**	**0.580 (0.410–0.819)**	**0.002**
Educational level (university and above to below*)	1.300 (0.706–2.396)	0.400	**0.305 (0.101–0.922)**	**0.035**	1.389 (0.844–2.286)	0.196	0.473 (0.153–1.467)	0.195
Jobs (direct contact to indirect contact with patients*)	1.411 (0.917–2.170)	0.117	1.737 (0.767–3.934)	0.185	1.052 (0.754–1.467)	0.766	1.097 (0.611–1.968)	0.757
Professional title (senior to below*)	**1.901 (1.143–3.162)**	**0.013**	**1.988 (1.124–3.517)**	**0.018**	**2.011 (1.259–3.214)**	**0.003**	1.530 (0.917–2.551)	0.103
Medical history (yes to no*)	1.332 (0.875–2.027)	0.181	1.534 (0.979–2.404)	0.062	0.853 (0.583–1.246)	0.410	0.761 (0.499–1.161)	0.205
History of immunosuppressive therapy (yes to no*)	2.063 (0.616–6.909)	0.240	1.536 (0.403–5.858)	0.530	0.167 (0.021–1.295)	0.087	0.198 (0.025–1.559)	0.124
Number of people living with (≥3 to <3 people living with)	1.038 (0.763–1.410)	0.813	0.987 (0.690–1.412)	0.943	1.099 (0.851–1.420)	0.469	1.317 (0.967–1.794)	0.081
Vaccination rate among people living with (≥50 to <50%)	**7.505 (5.479–10.280)**	**<0.001**	**7.890 (5.614–11.089)**	**<0.001**	1.225 (0.929–1.616)	0.151	1.098 (0.812–1.484)	0.544
Flu-like symptom experience (yes to no*)	0.930 (0.697–1.241)	0.623	0.855 (0.628–1.163)	0.318	**1.549 (1.211–1.982)**	**<0.001**	**1.523 (1.164–1.992)**	**0.002**
Willingness to be vaccinated (yes to no*)	**1.628 (1.218–2.177)**	**0.001**	**1.514 (1.110–2.066)**	**0.009**	–	–	–	–

OR, Odds-ratio. ^a^Adjusted for age, gender, educational level, and professional title. University and above includes bachelor’s degree, master’s degree, and doctorate or above; below includes junior college and below. Direct-contact jobs include nurses, medical professionals, medical technicians, and pharmacists; indirect-contact jobs include healthcare recruiters, managers/administrative roles, researchers, project-based staff, and other support roles. Bold values show the statistically significant results.

For willingness to be vaccinated, female HCWs were significantly less likely than males to express willingness (adjusted OR: 0.58, 95% CI: 0.41–0.82, *p* = 0.002). Moreover, HCWs who had experienced flu-like symptoms in the past 3 months were more inclined toward future vaccination (adjusted OR: 1.52, 95% CI: 1.16–1.99, *p* = 0.002). Other factors, including age, educational level, and medical history, were not significantly associated with vaccine uptake or willingness after adjustment (*p* > 0.05; Table 6).

## 4 Discussion

This cross-sectional study examined the uptake, willingness, and influencing factors surrounding seasonal influenza vaccination among HCWs in a tertiary hospital in Xi’an, China, during the post-COVID-19 era. Because this was a voluntary, institution-based survey conducted in a single tertiary hospital, the sample was not designed to be statistically representative of the healthcare workforce in Xi’an, Shaanxi Province, or China as a whole. While respondents were drawn from multiple departments and job categories, the distribution reflects the workforce structure of this single tertiary hospital. Accordingly, our estimates should be interpreted as reflecting HCWs working in a large tertiary-hospital context with almost similar organizational structures, occupational mix, and vaccine-delivery arrangements.

The results demonstrated that although HCWs exhibit awareness and favorable attitudes toward vaccination, the actual uptake is low and has shown a decreasing trend following the COVID-19 pandemic. These findings highlight complicated individual, institutional, and systemic factors and align with global trends, especially in low- and middle-income countries (31–33) (LMICs). Interpreting our findings through these behavioral frameworks helps clarify why uptake remained low despite general awareness. In the 3Cs model, safety concerns and distrust map to reduced *confidence*, perceiving influenza vaccination as unnecessary reflects *complacency* (low perceived need), and logistical barriers (e.g., time and access constraints) reflect limited *convenience*. Consistent with HBM constructs, recent flu-like symptoms may function as a cue to action and increase perceived susceptibility, thereby strengthening willingness. Similarly, the strong association between household vaccination rates/peer influence and HCWs’ uptake is consistent with the role of social norms (TPB) in shaping intention and behavior. This framework-based interpretation supports multi-component interventions that simultaneously address confidence (transparent safety communication), complacency (risk/benefit messaging), and convenience (on-site or department-based delivery).

According to our survey, only 19.65% of HCWs said they have received at least one influenza vaccination in the previous 2 years. This number is in line with previous studies from China, where vaccination rates for HCWs have varied from 5 to 25.3% depending on region and policy support (12, 34). This is significantly lower than vaccination coverage in high-income nations, such as the United States, where rates exceed 75% among HCWs due to institutional mandates and well-coordinated immunization programs (35).

Furthermore, our results showed a significant decline in vaccination rates from 2022 to 2023, reversing gains observed during the COVID-19 pandemic. This trend raises the possibility that the increased vaccination uptake during the pandemic was temporary and did not last as public urgency subsided. Similar “post-pandemic declines” have been noted in other settings, often attributed to vaccine fatigue, diminished public interest, and reduced institutional emphasis once pandemic controls (36, 37).

HCWs who lived in households with a higher vaccination rate were significantly more likely to have received their own vaccinations, according to multivariable regression analysis. This indicates the role of peer and family influence. This finding is consistent with previous research demonstrating that social norms and group behaviors play a key role in vaccine decision-making among both the general public and HCWs (38). Moreover, HCWs who expressed willingness to be vaccinated demonstrated higher odds of actual vaccine uptake, emphasizing the predictive value of attitudinal intention.

HCWs holding senior professional titles were more likely to have been vaccinated. This may reflect greater risk awareness, stronger adherence to institutional recommendations, and easier access to occupational health services among senior staff (39).

Moreover, those with recent flu-like symptoms were remarkably more willing to be vaccinated in the future, perhaps as a result of their perception of their increased vulnerability or severity of illness, a factor highlighted in the Health Belief Model (40).

In contrast to earlier research that found women generally report higher vaccination uptake, in our survey, gender differences were noted, with female healthcare workers being less likely than males to express willingness. This could be a result of regional sociocultural dynamics, disparities in the roles of caregivers, or concerns about the safety of vaccines for female reproductive health, which have been raised in previous studies (41, 42).

As per global research, vaccine hesitancy emerged as a significant obstacle. Concerns regarding vaccine safety and side effects were the most frequently stated reasons (66.18%), followed by opinions that vaccination was unnecessary (44.22%). These results are consistent with the WHO’s 3Cs model (confidence, complacency, convenience), which identifies necessity and vaccine safety as key factors influencing hesitancy (18). Furthermore, 15.92% of respondents questioned the vaccine’s efficacy, emphasizing concerns that seasonal influenza vaccines offer variable protection due to annual antigenic drift. Transparent risk communication and frequent updates on vaccine efficacy are necessary to reduce these concerns, which are widespread throughout the world (43).

Although access-related barriers were less frequently cited, but still present (8.53%), indicating a need for improvement in institutional delivery mechanisms. User-friendly access points are crucial because they have been demonstrated to boost uptake (32). This is further supported by the stated preference for department-based vaccination, mobile clinics, and enhancing access and logistics (44).

The study’s findings have several implications for public health initiatives. First, encouraging influenza vaccination among HCWs requires an extensive approach that combines structural interventions (e.g., on-site vaccination, mobile services), behavioral strategies (e.g., targeted education), and financial support (e.g., subsidies for HCWs and their families). In addition, recommendations for including family members in vaccination programs reflect an opportunity to extend protective circles and benefit from familial influence.

Second, institutional management should prioritize clear and early communication strategies. Our study found that 14.59% of HCWs specifically asked for more robust awareness campaigns. Reminders, educational seminars, and senior staff role modeling are effective ways to increase uptake, according to studies conducted elsewhere (45, 46).

Last but not least, our findings show that long-term plans are required to maintain the momentum for vaccinations after a crisis like COVID-19. Long-term change necessitates integrating vaccination into healthcare culture, in line with WHO recommendations for routine HCW immunization policies, even though pandemic periods may momentarily increase uptake (47).

### 4.1 Strengths and limitations

This study used a large and diverse sample of HCWs from a major tertiary hospital in western China, enhancing generalizability within similar institutional settings. The comprehensive variable analysis (including demographic, clinical, and attitudinal variables) strengthens the robustness of the findings. The inclusion of both quantitative and qualitative components (i.e., open-ended suggestions) provided valuable insights into HCWs’ perspectives and practical barriers.

However, this study was limited to one hospital, which may not reflect trends in rural settings or smaller institutions or even other tertiary hospitals across China, as vaccination policies, occupational-health services, organizational culture, and regional vaccine availability can differ substantially across institutions and provinces. Our sample’s occupational and sex composition reflects a tertiary-hospital workforce (e.g., a large nursing component and a high proportion of female staff), which is broadly consistent with national patterns of a female-majority health workforce but may still limit comparability with settings that have different staffing structures. Therefore, multi-center studies using stratified sampling and weighting to regional workforce distributions are needed to improve generalizability.

In addition, we did not collect a dedicated measure of employment arrangement (e.g., formally employed vs. contract/project-based). Employment status may influence institutional access (e.g., eligibility for subsidized vaccination), occupational health policies, scheduling flexibility, and perceived organizational support, all of which can shape vaccination uptake and willingness. Future multi-center studies should explicitly stratify by employment arrangement to assess its independent contribution.

Self-reported data introduces risks of recall bias and social desirability bias, especially regarding vaccine uptake and willingness. In addition, the cross-sectional design prevents causal inferences. Longitudinal studies would be required to evaluate changes in attitudes and behaviors over time. Although the response rate was high, we lacked complete information on non-respondents, which may affect representativeness.

## 5 Conclusion

Despite the well-established benefits of influenza vaccination among healthcare workers, uptake remains suboptimal in China, with a notable decline after the COVID-19 pandemic. Our findings underscore the importance of peer and family influence, recent flu-like symptoms, and attitudinal willingness as key predictors of vaccination behavior. However, safety concerns, doubts about necessity, and logistical barriers remain prevalent and must be addressed through multi-pronged strategies. To improve vaccination coverage, health institutions should consider enhancing accessibility, offering free or subsidized vaccines, and implementing comprehensive educational campaigns tailored to HCWs’ concerns. Targeting vaccine hesitancy with evidence-based communication and facilitating convenient vaccination models may contribute to more sustainable increases in coverage. Given the role of HCWs as both beneficiaries and promoters of vaccination, improving their uptake is not only a matter of occupational health but a critical step toward strengthening public trust and pandemic preparedness in China’s healthcare system.

## Data Availability

The raw data supporting the conclusions of this article will be made available by the authors, without undue reservation.
